# Frequency, Local Dynamics, and Genomic Characteristics of ESBL-Producing *Escherichia coli* Isolated From Specimens of Hospitalized Horses

**DOI:** 10.3389/fmicb.2021.671676

**Published:** 2021-04-16

**Authors:** Anne Kauter, Lennard Epping, Fereshteh Ghazisaeedi, Antina Lübke-Becker, Silver A. Wolf, Dania Kannapin, Sabita D. Stoeckle, Torsten Semmler, Sebastian Günther, Heidrun Gehlen, Birgit Walther

**Affiliations:** ^1^Advanced Light and Electron Microscopy (ZBS-4), Robert Koch Institute, Berlin, Germany; ^2^Genome Sequencing and Genomic Epidemiology (MF2), Robert Koch Institute, Berlin, Germany; ^3^Centre for Infection Medicine, Institute of Microbiology and Epizootics, Freie Universität Berlin, Berlin, Germany; ^4^Equine Clinic, Surgery and Radiology, Freie Universität Berlin, Berlin, Germany; ^5^Institute of Pharmacy, Universität Greifswald, Greifswald, Germany

**Keywords:** horse, ESBL, *Escherichia coli*, antibiotic resistance, multidrug resistant, spread

## Abstract

Previous research identified veterinary clinics as hotspots with respect to accumulation and spread of multidrug resistant extended-spectrum β-lactamase (ESBL)-producing *Escherichia coli* (EC). Therefore, promoting the prudent use of antibiotics to decrease selective pressure in that particular clinical environment is preferable to enhance biosecurity for animal patients and hospital staff. Accordingly, this study comparatively investigated the impact of two distinct perioperative antibiotic prophylaxis (PAP) regimens (short-term versus prolonged) on ESBL-EC carriage of horses subjected to colic surgery. While all horses received a combination of penicillin/gentamicin (P/G) as PAP, they were assigned to either the “single-shot group” (SSG) or the conventional “5-day group” (5DG). Fecal samples collected on arrival (t_0_), on the 3rd (t_1_) and on the 10th day after surgery (t_2_) were screened for ESBL-EC. All isolates were further investigated using whole genome sequences. In total, 81 of 98 horses met the inclusion criteria for this study. ESBL-EC identified in samples available at t_0_, t_1_ and t_2_ were 4.8% (SSG) and 9.7% (5DG), 37% (SSG) and 47.2% (5DG) as well as 55.6% (SSG) and 56.8% (5DG), respectively. Regardless of the P/G PAP regimen, horses were 9.12 times (95% CI 2.79–29.7) more likely to carry ESBL-EC at t_1_ compared to t_0_ (*p* < 0.001) and 15.64 times (95% CI 4.57–53.55) more likely to carry ESBL-EC at t_2_ compared to t_0_ (*p* < 0.001). ESBL-EC belonging to sequence type (ST) 10, ST86, ST641, and ST410 were the most prevalent lineages, with *bla*_*CTX*__–__*M*__–__1_ (60%) being the dominant ESBL gene. A close spatio-temporal relationship between isolates sharing a particular ST was revealed by genome analysis, strongly indicating local spread. Consequently, hospitalization itself has a strong impact on ESBL-EC isolation rates in horses, possibly masking differences between distinct PAP regimens. The results of this study reveal accumulation and spread of multi-drug resistant ESBL-EC among horses subjected to colic surgery with different P/G PAP regimens, challenging the local hygiene management system and work-place safety of veterinary staff. Moreover, the predominance of particular ESBL-EC lineages in clinics providing health care for horses needs further investigation.

## Introduction

The occurrence of zoonotic and multidrug resistant (MDR) pathogens, such as methicillin resistant *Staphylococcus aureus* (MRSA), *Acinetobacter* spp. and extended-spectrum β-lactamase (ESBL)-producing Enterobacterales, are an ongoing challenge to both, biosecurity and hygiene in veterinary clinics (Walther et al., 2017, 2018a; Royden et al., 2019). Previous studies reported the constant admission of equine patients carrying ESBL-producing *Escherichia coli* (ESBL-EC) to horse clinics (Apostolakos et al., 2017; Walther et al., 2018b). Moreover, hospital associated infections (HAI) have been linked to local spread of the above-mentioned MDR pathogens in horse clinics (Walther et al., 2014b; van Spijk et al., 2019). These infections are often difficult to handle due to a multitude of additional antimicrobial resistances (AMR) commonly associated with ESBL-EC (Wieler et al., 2011). In recent years, reports on ESBL-EC carriage and fecal shedding in horses have increased (de Lagarde et al., 2019; Hordijk et al., 2020). Hospital stay alone, for instance, as well as antibiotic courses have only recently been identified as important risk factors for ESBL-EC colonization of equine patients (Schoster et al., 2020).

So far, knowledge on ESBL-EC isolation rates and local dynamics considering particular equine patient groups, i.e., hospitalized horses subjected to colic surgery, is scarce. A combination of penicillin and gentamicin (P/G) is among the most common medications for perioperative antibiotic prophylaxis (PAP) in horse surgery, including operative abdominal (colic) interventions (Dallap Schaer et al., 2012; Teschner et al., 2015). While different guidelines for antibiotic administration in horses strongly suggest to abstain from PAP prolongation beyond 24 h after elective interventions (Weese et al., 2008; Swedish Veterinary Association, 2013), horses subjected to abdominal surgery seem to commonly receive the P/G combination for 5 days afterward (Teschner et al., 2015). In a previous study, reduction of the P/G PAP to 72 h (3 days) after surgery showed no significant influence on the clinical outcome of the equine patients (Dallap Schaer et al., 2012). However, the putative effect of decreased selective (antibiotic) pressure on the local MDR pathogen load, especially ESBL-EC, was not investigated in the mentioned study.

This study was carried out within an interdisciplinary research network (Köck et al., 2020) investigating strategies to enhance the prudent use of antibiotics in veterinary medicine. Accordingly, the aims were (i) to assess ESBL-EC phenotypes and isolation rates in hospitalized horses, (ii) to investigate whether a short-term or a prolonged P/G PAP influences these rates, and (iii) to identify potential ESBL-EC transmission events and local dynamics.

## Materials and Methods

### Ethics and Study Outline

According to the German regulation authorities for research with animal subjects, the comparison of two PAP regimens does not require approval (Landesamt für Gesundheit und Soziales, Berlin, 18.04.2017). Written owner’s consent with respect to involvement of their horses in the study was obtained directly during the hospital admission process.

**FIGURE 1:**
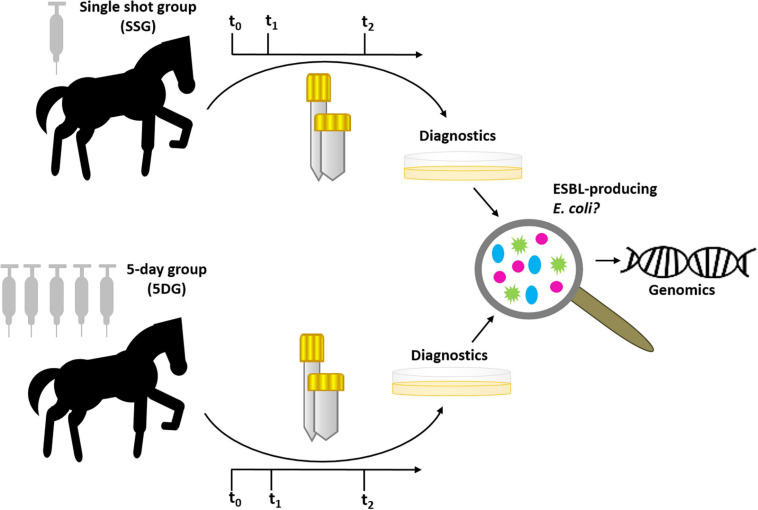
Graphical abstract of the prospective study. Horses were assigned to either the “single-shot group” (SSG) or the “5-day group” (5DG) per lot (sealed envelope drawn directly at hospital admission). Fecal samples and nostril swabs were collected at t_0_ (hospital admission), t_1_ (3 days after surgery) and t_2_ (10 days after surgery). All samples were screened for putative ESBL-EC, subjected to the confirmatory testing followed by AST and whole genome sequencing.

Here, we have comparatively investigated the effect of two distinct PAP regimes on isolation rates of ESBL-EC from specimens of horses subjected to colic surgery. A graphical abstract of the study design is provided in Figure 1. Horses included in this study were assigned per lot (sealed envelope drawn at the day of surgery) to one of two groups: the “5-day group” (5DG) which received a combination of penicillin (22,000 IU/kg q 6 h) and gentamicin (6.6 mg/kg q 24 h) (short: P/G) (Dallap Schaer et al., 2012; Teschner et al., 2015) for 5 days and the “single-shot group” (SSG), which received P/G PAP only directly before, and, if necessary due to prolonged surgery- also during the elective procedure Briefly, horses considered as study participants needed to be older than 1 year, had to be clear of any clinical signs of infectious diseases before the intervention and had to recover from anaesthesia (“stand up”) after surgery. Furthermore, equine patients were excluded from further participation (i.e., consideration of specimens at t_1_ and/or t_2_; Figure 1) when their hospital stay ended prematurely due to euthanasia (premature), discharge and/or if the antibiotic regime they had been assigned to was no longer strictly followed, regardless of the particular reasons for these interventions. In consequence, the number of valid specimens available per time point shrank toward the end of the study.

### Sample Collection

In order to investigate ESBL-EC rates in specimens from the equine patients, fecal samples and nostril swabs (Copan Liquid Amies Elution Swab, flocked) were directly collected from each horse at the time of hospital admission (t_0_) as described previously (Walther et al., 2018b). A second and a third sampling was carried out on day three (t_1_) and ten (t_2_) after surgery (Figure 1). All specimens were stored at 4°C and subjected to microbiological diagnostics latest on the next day. We included the nostril sampling in this study, since dust particles containing ESBL-EC have been reported as a potential source of contamination and, possibly, colonization in farmers exposed to an environment prone to MDR pathogens (Fischer et al., 2017).

### Bacterial Identification and Antimicrobial Susceptibility Testing

All fecal samples and nostril swabs were initially cultured on Brilliance^TM^ ESBL Agar plates (Thermo Scientific^TM^, Germany) overnight. Colonies showing characteristic growth signatures of ESBL-EC on chromogenic screening plates were further investigated. In case of distinct phenotype appearances of presumptive EC growing on the plates, all isolates were subjected to the ESBL confirmatory test according to the Clinical and Laboratory Standards Institute (CLSI) recommendations (Clinical and Laboratory Standards Institute [CLSI], 2018). Additionally, disk diffusion method was performed to detect putative plasmidal AmpC (pAmpC)-producers using cefoxitin (30 μ) with a ≤18 mm screening cut-off (Polsfuss et al., 2011). Species confirmation was achieved by Matrix-Assisted Laser Desorption/Ionization-Time Of Flight (MALDI-TOF) mass spectrometry (Bruker, Germany). The Vitek2 AST-GN38 and AST-GN 96 cards (bioMérieux, Germany) were used for antimicrobial susceptibility testing (AST) on the VITEK^®^2 system (BioMérieux, Germany) including enrofloxacin, gentamicin, trimethoprim-sulfamethoxazole and tetracycline following CLSI standards (Clinical and Laboratory Standards Institute [CLSI], 2020).

### Whole Genome Sequencing, Antimicrobial Resistance Gene Detection and Phylogenetic Relationship of the Isolates

DNA isolation was performed for all confirmed ESBL/pAmpC-EC with the QIAamp^®^ DNA Mini Kit (250) (Qiagen, United States) and the respective DNA was subsequently stored at −20°C. Whole genome sequencing (WGS) was performed using Illumina MiSeq 250 bp paired-end sequencing with an obtained coverage >80. After adapter trimming, 247.9 bp remained on average per read. Illumina raw read data sequenced for this study is available at National Center for Biotechnology Information (NCBI) under Bioproject ID: PRJNA698802.

Assembled draft genomes of the isolates were obtained from SPAdes v.3.13.1 (Bankevich et al., 2012) and annotated with Prokka v.1.14.6 (Seemann, 2014). WGS data were used for the determination of the sequence type (ST) and resistance genes (threshold: 98% ID, 90% minimum query coverage) performed by Center for Genomic Epidemiology (Bortolaia et al., 2020). PlasmidFinder and pMLST v.1.3 pipelines were used for the identification of plasmids associated with Enterobacteriales (Zankari et al., 2012; Carattoli et al., 2014). A core genome alignment was calculated using Roary v.3.13.0 (Page et al., 2015), with a minimum sequence identity of 95%. The alignment was used to construct a maximum likelihood-based phylogeny with RAxML v.8.2.10 (Stamatakis, 2014) and 100 bootstraps under the assumption of the gtr-gamma DNA substitution model.

In order to investigate potential MDR pathogen transmission events within the horse clinic, pairwise single-nucleotide polymorphism (SNP) distances were calculated with snp-dists v.0.7.0^1^ and visualized using the R package “pheatmap”^2^. Furthermore, pairwise SNP distances were utilized to identify clonally related ESBL-EC genomes for each of the major sequence type complexes (STC) identified (Supplementary Figures 1–3). Since the mutation rate for ESBL-EC genomes was estimated to be 4.14 × 10^–7^ SNPs per site per year (Ludden et al., 2020), a clonal relationship was defined conservatively as isolates with five or fewer SNPs between any two members of a group, as reported previously (D’Souza et al., 2019).

### Statistical Analysis

Statistical analyses were conducted using the SPSS software (Statistical Package of Social Science, version 26.0, Chicago, IL, United States). The comparative analysis of the proportion of ESBL-EC positive equine patients was performed using the McNemar test for categorial variables and by analyzing the proportions of ESBL-positive fecal samples at two sampling time points (t_0_ and t_1_) or (t_0_ and t_2_). Generalized estimating equation (GEE) analysis for clustered binomial responses was used to determine the effect of hospitalization (t_1_ and t_2_), different P/G PAP regimes (short-term or prolonged), and a potential interaction effect, on the occurrence of ESBL-EC in fecal samples of horses subjected to colic surgery. A *p*-value of < 0.05 was considered statistically significant.

## Results

### Study Population and Sample Acquisition

**FIGURE 2:**
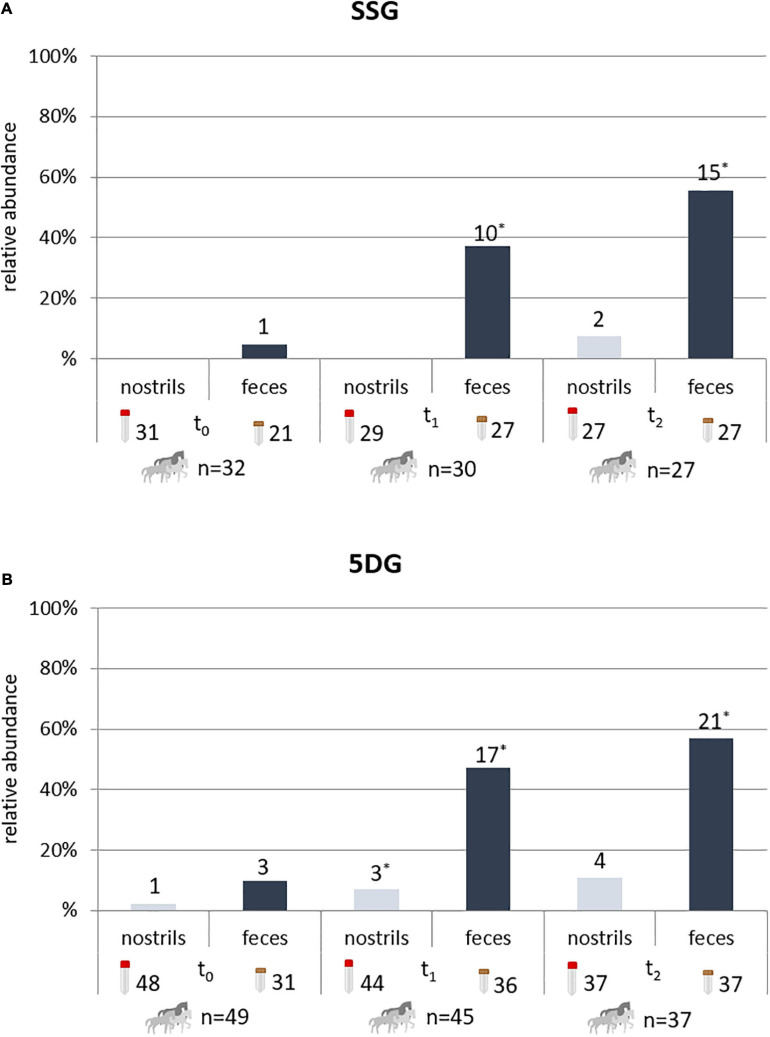
Horses with ESBL-EC-positive specimens during hospital stay. Horses with ESBL-EC-positive specimens (nostril swabs and fecal samples) at t_0_ (hospital admission), t_1_ (3 days after surgery) and t_2_ (10 days after surgery). Since defecation disorders are frequently associated with colic syndrome, fecal samples were not available for all horses at every time point. For the single-shot group (SSG), 32 horses met the criteria for participation in this study at t_0_ **(A)**, with 31 nostril swabs and 21 fecal available for the screening procedure. Of the 49 horses initially allotted to the 5-day group (5DG), 48 nostril swabs and 31 fecal samples were collected at t_0_ **(B)**. Due to the strict study design, specimens of horses failing the inclusion criteria in the ongoing study were excluded from further consideration at t_1_ and/or t_2_, resulting in 27 participating horses for the SSG and 37 for the 5DG at t_2_. *, more than one isolate/specimen possible.

A total of 98 horses subjected to abdominal surgery due to acute abdomen (colic) from January 2018 to February 2020 have been considered as study participants. However, only 81 of these horses met the inclusion criteria for this study at t_0_, with *n* = 32 belonging to the SSG and *n* = 49 to the 5DG (Figure 2). Due to the medical nature of colic syndrome, which is often accompanied by defecation disorders, samples were not available for all horses at all sampling times, as indicated by the numbers associated with the sample symbols in Figure 2. Moreover, until the end of the study, samples from five horses belonging to the SSG and 12 from the 5DG were excluded from further evaluation—the reasons included sudden discharge, euthanasia due to animal welfare or deviation from the antibiotic protocol. In addition, temporary unavailability of Benzylpenicillin for veterinary use caused premature termination of the study in 02/2020. This event lead to a restricted number of study participants and therefore also limited the representativeness of the study outcome.

### Isolation Rates of ESBL-EC in Specimens of Equine Patients Subjected to Colic Surgery Receiving Different Regimes of P/G PAP

Considering the isolation rates for each sampling point, ESBL-EC were occasionally identified in nostril swabs obtained from both horse groups investigated (Figure 2 and Supplementary Table 1). In total, 2/87 (2%) nostril swabs obtained from the SSG and 8/129 (6%) of the 5DG were ESBL-EC positive.

In the SSG, 1/21 (4.8%) fecal samples collected at the time of hospital admission (t_0_) were positive for ESBL-EC, while the isolation rate increased to 10/27 (37%) at t_1_. At the tenth day of hospital stay, 15/27 (55.6%) of the SSG fecal samples were positive for ESBL-EC (*p* = 0.003). The ESBL-EC rate among samples from horses belonging to the 5DG was 3/31 (9.7%) at t_0_ and increased to 17/36 (47.2%) at t_1_. At t_2_ (i.e., the tenth day after surgery and the fifth day after the last antibiotic course), 21/37 (56.8%) fecal samples were ESBL-EC positive (*p* = 0.013; Figure 2). There were no statistically significant differences between the proportions of ESBL-EC positive fecal samples of the two study groups investigated (Figure 2). Regardless of the P/G PAP regime, horses were 9.12 times (95% CI 2.79–29.7) more likely to carry ESBL-EC at t_1_ compared to t_0_ (*p* < 0.001) and 15.64 times (95% CI 4.57–53.55) more likely to carry ESBL-EC at t_2_ compared to t_0_ (*p* < 0.001).

### Antibiotic Susceptibility Profiles and Antimicrobial Resistance Genes

AST results for the most common antibiotics in equine medicine (enrofloxacin, gentamicin, trimethoprim-sulfamethoxazole and tetracycline) obtained for 85 ESBL-EC are shown in Table 1. Of note, 75 (88%) of the 85 investigated ESBL-EC isolates showed resistance toward aminoglycoside gentamicin and 78 (92%) toward the sulfonamide-trimethoprim combination. Eight isolates were ESBL-producers only, two isolates showed resistance toward one further class of antimicrobials, 24 toward two additional classes, 32 toward three additional classes and 3 toward antimicrobials belonging to four additional substance classes (Table 1).

**TABLE 1:** AST profiles of ESBL-EC isolated from specimens of hospitalized horses.

ST	n	ENR	GEN	SXT	TET
**10**	12	≤0.12	**≥16**	**≥320**	**≥16**
	1	≤ 0.12	≤1	≤20	≤1
	2	**1**	**≥16**	**≥320**	≤1
	1	**0.5**	**≥16**	**≥320**	**≥16**
	1	**0.5**	**≥16**	**≥320**	≤1
	2	**≥4**	**≥16**	**≥320**	**≥16**
**1,245**	5	≤0.12	**≥16**	**≥320**	=1
**1,250**	2	≤0.12	**≥16**	**≥320**	≤1
	1	≤0.12	**≥16**	**≥320**	=16
	1	**0.5**	**≥16**	**≥320**	≤1
	1	≤0.12	≤1	**≥320**	≤1
**224**	5	**≥4**	**≥16**	**≥320**	**≥16**
**410**	3	**≥4**	**8**	**≥320**	=16
	4	**≥4**	**≥16**	**≥320**	**≥16**
	2	**0.5**	**8**	**≥320**	**≥16**
	1	≤0.12	≤1	≤20	≤1
**641**	1	≤0.12	**≥16**	**≥320**	≤1
	6	≤0,12	**≥16**	**≥320**	**≥16**
	1	**0.5**	**≥16**	**≥320**	≤1
	1	**≥4**	**≥16**	**≥320**	**≥16**
**86**	2	**1**	**≥16**	**≥320**	**≥16**
	6	**1**	**≥16**	**≥320**	**≥16**
**155**	2	≤0.12	**≥16**	**≥320**	≤1
**1,686**	1	≤0.12	**≥16**	**≥320**	**≥16**
	1	**1**	=16	**≥320**	≤1
**1,730**	1	**0.5**	≤1	≤20	≤1
	1	**1**	≤1	≤20	≤1
**2,035**	1	**1**	**≥16**	**≥320**	≤1
**2,325**	1	**1**	≤1	≤20	≤1
**2,350**	1	≤0.12	**≥16**	≤20	≤1
	1	**0.5**	**≥16**	**≥320**	**≥16**
**617**	3	**≥4**	**≥16**	**≥320**	**≥16**
**648**	1	**≥4**	**≥16**	**≥320**	**≥16**
**6,589**	1	**0.5**	**≥16**	**≥320**	≤1
**7,459**	1	≤0.12	≤1	≤20	≤1
**453**	1	0.25	≤1	≤20	≤1
**973**	1	≤0.12	≤1	≤20	≤1
**NA**	1	**1**	≤1	**≥320**	**≥16**
**Novel ST**	1	**1**	**≥16**	**≥320**	**≥16**
**NA**	1	≤0.12	**≥16**	**≥320**	**≥16**
**1,204**	1	**1**	**≥16**	**≥320**	≤1
**NA**	1	**0.5**	**≥16**	**≥320**	≤1
**1,709**	1	≤0.12	**≥16**	**≥320**	=1

*Antimicrobial susceptibility testing (AST) results of ESBL-producing E. coli. In order to assess the importance of the AST profiles with respect to therapy options in case of infection, plain numbers indicate susceptibility and bold ones a resistant phenotype. ST, sequence type; n, number of isolates; ENR, enrofloxacin; GEN, gentamicin; SXT, trimethoprim-sulfamethoxazole; TET, tetracycline.*

**FIGURE 3:**
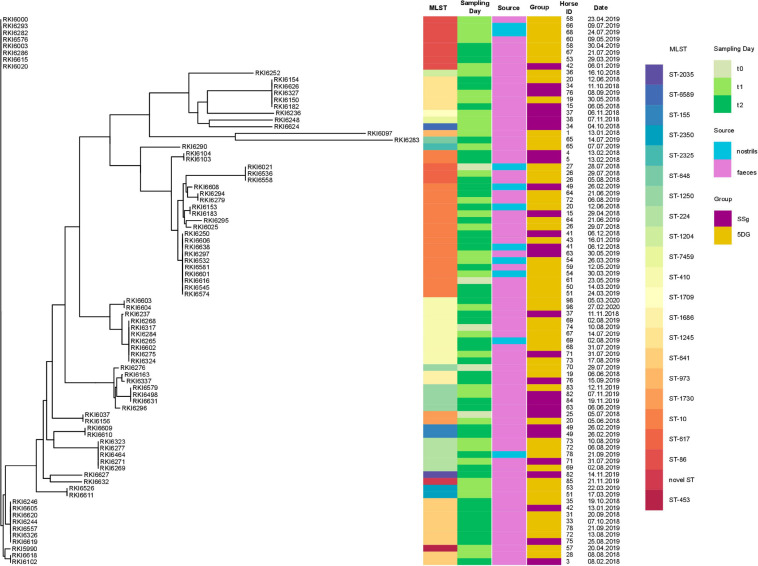
Core-genome based phylogeny of 82 equine ESBL-EC isolated from hospitalized horses subjected to colic surgery. Maximum likelihood phylogenetic tree generated with RAxML and visualized using the CLC Genomics Workbench based on 2,700 orthologous genes from 82 ESBL-EC WGS. Sequence type (MLST), sampling day (t_0_, t_1_, or t_2_), individual horses (ID), sampling date, sampling site (source) and study group (SSG or 5DG) are indicated.

Unfortunately, three isolates were, by mistake, not stored and were therefore not included in the further WGS-based analysis process. Genes conferring β-lactam resistance and other antimicrobial resistances (Supplementary Table 1) identified in the remaining 82 whole genome sequences were in concordance with the AST profiles (Supplementary Table 1). The most predominate beta-lactam resistance gene identified in this study was *bla*_*CTX* – *M* – 1_ (*n* = 49), followed by *bla*_*CTX*–*M*–15_ (*n* = 13), *bla*_*SHV*–12_ (*n* = 11), *bla*_*OXA*–1_ (*n* = 7) and *bla*_*CTX*–*M*–14_ (*n* = 5). One isolate carried *bla*_*CTX*–*M*–3_ (*n* = 1) and the broadened β-lactam resistance was conferred by pAmpC (*bla*_*CMY*–2_) in one case (Figure 3 and Supplementary Table 1). Aminoglycoside resistance genes were most frequently associated with ESBL-EC (74/82 genomes, 90%), especially *aac* (3) variants (67 genomes, 82%), followed by genes enhancing resistance toward sulfonamides (*sul*1 and/or *sul*2, 89%) and trimethoprim (*dfr*A and *dfr*G genes, 88%). More details on the distribution of antimicrobial resistance genes in ESBL-EC isolates are provided in Supplementary Table 1.

### Phylogenetic Background of ESBL/pAmpC-EC Isolated From Hospitalized Horses

A phylogenetic tree was generated from 2,700 orthologs genes identified using WGS data (Figure 3). Based on WGS analysis, 21 different STs were identified (Supplementary Table 1 and Figure 3), including STs belonging to three dominating STC, i.e., STC10 (ST10; 23%), STC23 (ST410, 12%), and STC86 including ST86 (10%), ST641 (11%), and ST453 (1.2%), revealing an overall broad heterogeneity of phylogenetic backgrounds for the ESBL/pAmpC-EC isolated in this study.

**FIGURE 4:**
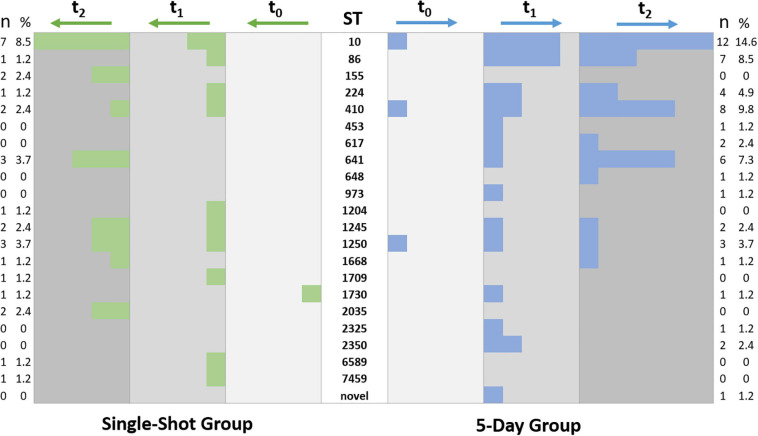
Occurrence and distribution of sequence types associated with equine ESBL-EC. Each colored square indicates an ESBL-EC, isolates of the SSG are represented in green and those from samples of the 5DG are blue. Sampling time points are indicated by different shades of gray and column headings.

STs associated with isolates from only one study group occurred rarely (e.g., ST453, *n* = 1, 5DG; ST617, *n* = 2, 5DG; ST2035, *n* = 2, SSG; ST1709, *n* = 1, SSG), a detailed illustration on the distribution of STs among the study groups is provided in Figure 4.

**FIGURE 5:**
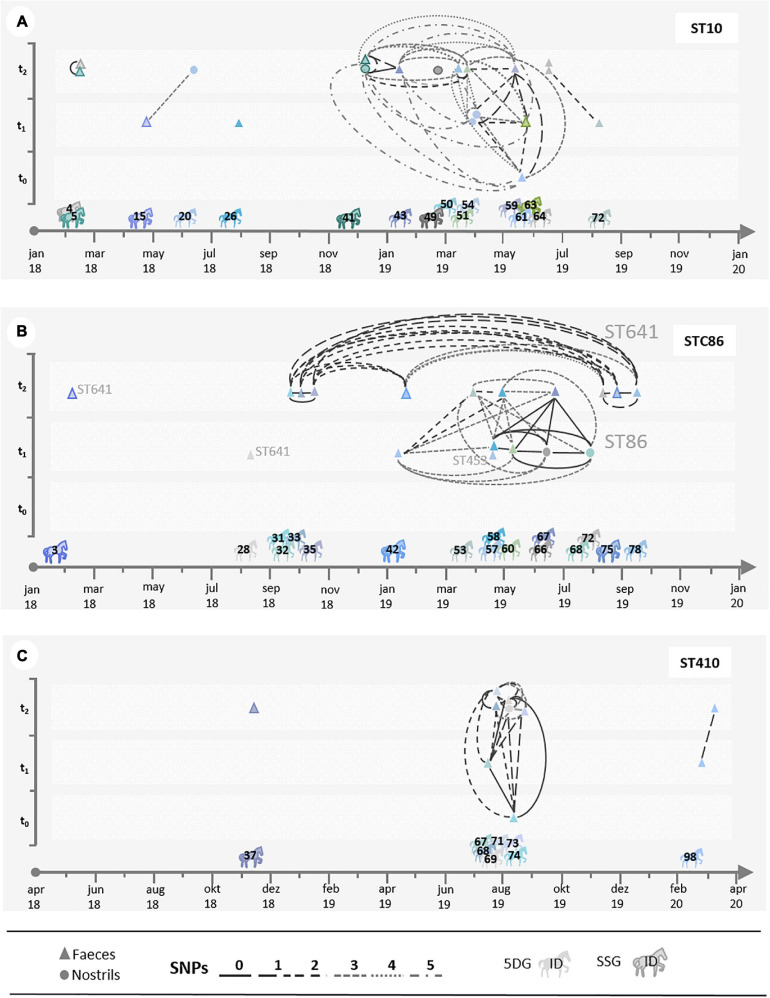
Local spread of distinct ESBL-EC. The illustrations **(A–C)** show scatter plots displaying SNP distances calculated for the most prevalent ESBL-EC lineages on a time scale. In order to assess the possible phylogenetic and temporal relationships between the isolates, isolates of each horse (indicated by its identification number (ID)) share a particular color. Isolates gained from nostril swabs are indicated by circles, fecal isolates by triangles. The number of SNPs between two isolates is marked with a specific line. **(A)** Shows all isolates belonging to ST10; **(B)** STC86 (ST641 and ST86); and **(C)** ST410.

Considering all ESBL-EC-positive nostril samples (*n* = 12; 5.5%), only one horse (54, Figure 3) was positive for ESBL-EC ST10 *bla*_*CTX*–*M*–1_ twice (t_1_ and t_2_). The frequency of ESBL-EC isolated from nostril swabs increased during hospitalization (t0 = 1; t1 = 4, t2 = 7: comment: some swabs were positive for more than one phenotype). The nostril swabs and fecal samples of the horses with ID41 (t_2_, ST10 *bla*_*CTX*–*M*–1_) and ID69 (t_2_, ST410 *bla*_*CTX*–*M*–15_) were positive for the same ESBL-EC clone (Figure 5). Contrary, the clonal background of the nostril/fecal isolates of horses with the IDs 20 (t_2_: ST10 *bla*_*CTX*–*M*–1/_ST1245 *bla*_*CTX*–*M*–14_), 49 (t2: ST10 *bla*_*CTX*–*M*–1/_ST155 *bla*_*CTX*–*M*–1_), and 78 (t2: ST-224 *bla*_*CTX*–*M*–1/_ST641 *bla*_*CTX*–*M*–1_) were different (Supplementary Table 1).

Overall, fecal samples collected from 18 horses (*n* = 7 from the SSG and *n* = 11 from the 5DG) were ESBL-EC positive at two different time-points (t_1_ and t_2_). In two cases, isolates from t_1_ and t_2_ differed by two SNPs (5DG, ID26, ST617 *bla*_*CTX*–*M*–15_ and 5DG, ID58, ST86 *bla*_*SHV*–12_) and were therefore considered as the same clone, respectively.

While all ST86 ESBL-EC were associated with *bla*_*SHV*–12_, distantly related ST1730 isolates were positive for *bla*_*SHV*–12_ as well. Further associations were obvious for *bla*_*CTX*–*M*–14_ with ST1245, while *bla*_*CTX*–*M*–15_ was identified in various clonal backgrounds (ST617, ST973, ST410, ST648, and ST2325) (Supplementary Table 1). Likewise, *bla*_*CTX*–*M*–1_ was distributed across multiple genomic lineages (Supplementary Table 1). *In silico* detection of plasmid replicons revealed variation for the number of replicons associated with the isolates as well as a broad range of different replicon types, including IncFIA/B and IncFII (Supplementary Table 1).

### Spatio-Temporal Dynamics of ESBL-EC

Since we noticed an increase of ESBL-EC isolation rates in specimens of horses belonging to both study groups during hospital stay, questions concerning spatio-temporal dynamics and persistence of distinct genomic lineages within the clinic (Figures 3, 4) have been raised. A scatter plot displaying SNP distances, calculated for the ESBL-EC genomes of those isolates belonging to the predominating STCs, was used to further investigate the suspected local spreads.

The illustration shows the time of hospital admission and the study group assignment for individual horses. ESBL-EC-positive samples at t_0_, t_1_, and t_2_ and close phylogenetic relationships with isolates obtained from specimens of other horses (Figures 5A–C) are indicated. The fecal sample of horse 5, for instance, was positive for an ST10 ESBL-EC at t_2_ with no recent history (i.e., t_0_, t_1_) of ESBL-positive specimens in our study (Figure 5A). In addition, an indistinguishable clone was identified in the t_2_-sample of horse 4, a horse which also had no ESBL-positive specimens before (Supplementary Table 1). Since the next ST10 ESBL-EC genome included here differed by at least 5,982 SNPs (Supplementary Figure 1), a common source of both isolates such as other (hospitalized) horses and/or the hospital environment seems likely, especially considering their close temporal relationship (Figure 5A).

Several additional close spatio-temporal relationships of ST10 ESBL-EC genomes are shown in Figure 5A. While there were “genomic singletons,” e.g., from samples of the horses 26 and 49, many further genomes were classified as closely related (Supplementary Figures 1–3), including indistinguishable genomes for isolates of the equine patients 41 and 43 (0 SNP difference); horse 54 and 59 (1 SNP difference) as well as horse 59 and 63 (2 SNP difference) and horse 64 and 72 (2 SNP difference). Considering isolation dates for isolates belonging to STC86, a direct or indirect link most likely exists between ST641 ESBL-EC obtained from fecal samples of horse 31 (t_1_) and 35 (t_2_). Moreover, a total of 7 closely related genomes (0–3 SNP difference) have been identified for ESBL-EC from specimens of horses hospitalized between 9/2018 and 9/2019 (Figure 5B and Supplementary Figure 2). A further cluster comprised 9 ST86 ESBL-EC from 8 horses, including 5 indistinguishable (0 SNP difference) genomes (Figure 5B). It should be noted that none of these horses were associated with an STC86 ESBL-EC specimen at hospital admission (Figure 5B). A cluster of 8 closely related (0–2 SNP difference) ST410 ESBL-EC genomes (STC23) was identified for specimens from 6 horses hospitalized in July and August 2019 (Figure 5C), once more strongly indicating a local and temporary spread and transmission of this particular clone as well.

## Discussion

This study showed that the proportion of fecal samples positive for ESBL-EC increases among hospitalized horses subjected to median laparotomy. It further provided evidence for the local spread of distinct MDR ESBL-EC clones and the local predominance of isolates belonging to ST10, STC86 and ST641 backgrounds. While the original intention of the present study was to comparatively investigate the particular influence of a short versus a prolonged P/G PAP regime on ESBL-EC isolation rates, the overwhelming effect of hospitalization seems to outmatch the (putative) differences between distinct P/G PAP courses.

### Isolation Rates of ESBL- EC in Specimens of Equine Patients

Caution is always needed when comparing study results with respect to study designs, population characteristics, local circumstances and methodical protocols used for ESBL (and pAmpC)-EC detection. In the present study, the observed ESBL-EC rates for fecal samples collected at hospital admission ranged from 5% (SSG) to 10% (5DG). Detected rates are in general concordance with previous studies, reporting proportions from 7 to 16% (Maddox et al., 2012; Walther et al., 2018b; Schoster et al., 2020), but lower than in studies using selective enrichment (Shnaiderman-Torban et al., 2020). A very recent study by Schoster et al. (2020) on hospitalized horses shedding ESBL-EC included different groups of equine patients while the duration of the PAP or anti-infective antimicrobial courses was determined individually for each patient by the veterinarian in charge. The fecal samples were collected -amongst other times- at hospital admission and 48–60 h afterward (Schoster et al., 2020). Interestingly, Schoster et al. (2020) found no significant difference concerning the ESBL-EC shedding rates in horses receiving either a fourth-generation cephalosporin or the common P/G combination. Although neither the study design nor the study population included by that particular work are directly comparable with the present study, interesting similarities are apparent: An increase in ESBL-EC detection rates reported for samples of hospitalized horses, for instance, are clearly in line with those recorded in the present study, since they have also noted an elevation in detection rates beyond 50% within 3 days after hospital admission. Schoster et al. (2020) also reported that the ESBL-EC isolation rates increased from 6.9 to 36% within 3 days after admission in horses which received no antibiotics at all. Consequently, a direct and overwhelming influence of hospitalization on the actual detection rate for samples from equine patients seems reasonable, probably also masking the effects of different P/G PAP regimes in the present study. However, since the ESBL-EC detection was lower in fecal samples obtained from the SSG, especially at t_1_ (Figure 2), a beneficial effect of less selective pressure associated with the short-term P/G PAP regime seems likely. Nonetheless, due to the limited number of study participants, the difference lacked statistical significance. A recent study on non-hospitalized horses identified antibiotic treatment and veterinary examinations as risk factors associated with ESBL-EC carriage (Kaspar et al., 2019). Further studies on the subject are clearly needed, especially since antimicrobial consumption was reported being among the most important risk factors for ESBL-EC colonization in human medicine (Harris et al., 2007).

One might argue that, if a single location is not sufficient to provide robust numbers of study participants, including additional clinics might enhance the study power. On the other hand, as recently shown by various authors, the local MDR burden tends to differ, especially during hospitalization (Apostolakos et al., 2017; Isgren et al., 2019; Hordijk et al., 2020; Shnaiderman-Torban et al., 2020), indicating a potential source of bias when considering multicenter studies. In addition, it seems likely that technical, structural and hygiene circumstances differ between facilities providing health care for horses, since overall comparability of standards, to the best of the authors’ knowledge, has not been achieved yet.

### Antibiotic Susceptibility Profiles and Antimicrobial Resistance Genes

In this study, the predominating ESBL-genes were *bla*_*CTX*–*M*–1_ (59.7%) and *bla*_*CTX*–*M*–15_ (15.8%), which supports earlier findings (Lupo et al., 2018; Walther et al., 2018b; Isgren et al., 2019; Hordijk et al., 2020).

Most of the isolates investigated in this study showed not only broadened resistance associated with their β-lactam resistance but also reduced susceptibility to other antimicrobial substances as well: Most of the ESBL-EC (51/85; 60%) isolates fulfilled the criteria for MDR (Schwarz et al., 2010). These MDR ESBL-EC pose therapeutic challenges for veterinarians since resistance genes conferring resistance to aminoglycosides, tetracyclines and trimethoprim/sulfamethoxazole are often present on the same plasmid (Li et al., 2019). Since the panel of antimicrobial substances currently available for approved administration to horses is very limited and includes the above mentioned antimicrobials (Maddox et al., 2012; Walther et al., 2014a), targeted strategies and guidelines are needed to cope with the apparent clinical challenges in equine antimicrobial therapy, especially to prevent hospital-associated infections and to reduce potential transmission between different sources and hosts, including humans (Walther et al., 2014b; Apostolakos et al., 2017; Royden et al., 2019; Hordijk et al., 2020).

### Phylogenetic Background of ESBL-EC Isolated From Samples of Hospitalized Horses

In total, 21 different STs have been identified for the ESBL-EC and the single pAmpC-EC, confirming earlier studies which reported a broad heterogeneity of phylogenetic backgrounds for ESBL-EC in horses (Apostolakos et al., 2017; Walther et al., 2018b; Schoster et al., 2020). Different ESBL-EC have been detected in a single sample, e.g., ESBL-EC belonging to ST10 and ST1245 in the “t_2_ fecal sample” of horse 20 (Supplementary Table 1). Hence, including different phenotypes in the ESBL-EC screening process is mandatory, as previously reported by Apostolakos et al. (2017). Considering the occurrence and broad distribution of STs among the isolate collection investigated (Figure 4), no obvious differences were identified.

However, ST10, STC86 (ST86, ST641, and ST453) and ST410 were the predominating phylogenetic lineages associated with the isolate collection, and assessment of their genomic relatedness revealed a close relationship (based on SNP differences) between many of the isolates belonging to a particular ST. Of note, neither ST86 nor ST641 were found to be associated with ESCL-EC isolated from specimens taken at hospital admission (Figure 5B).

Consequently, a common source and/or direct or indirect transmission of these ESBL-EC (Figure 3) seems more likely than spread of certain plasmids carrying β-lactam resistance genes, as previously suggested (Walther et al., 2018b). This hypothesis is supported by the finding that the overall diversity of genetic backgrounds seems to have decreased over time: while 18 different STs were associated with the 35 isolates obtained at t_1_, the 45 isolates representing t_2_ belonged to only 12 different STs, with ST10 (12 isolates), ST641 (8 isolates), and ST410 (6 isolates) being dominating lineages. Moreover, previous reports from Germany, the Netherlands and the United Kingdom also described ESBL-EC belonging to ST10, STC86, and ST410 for samples of horse origin as among the predominating lineages (Apostolakos et al., 2017; Walther et al., 2018b; Bortolami et al., 2019; Schoster et al., 2020).

Of note, MDR ESBL-EC belonging to ST10 were only recently reported for samples of meat, poultry and wildlife origin as well as for clinical human samples in Spain, once more emphasizing that this lineage is able to adapt to different life circumstances while crossing the borders of different niches and hosts (Diaz-Jimenez et al., 2020). Similar to the ubiquitous occurrence of *E. coli*-ST10, *E. coli* belonging to ST410 have also been described for samples from various hosts, including companion animals, livestock animals, wildlife- and human samples (Falgenhauer et al., 2016; Fischer et al., 2017; Reynolds et al., 2019). Moreover, ST410 was previously described as a genomic lineage containing high-risk trans-sectoral transmissible and multidrug-resistant clones in veterinary as well as human medicine (Schaufler et al., 2016; Roer et al., 2018). A recent study by Touchon et al. (2020) found that phylogeny and habitat shape the genetic diversification of *E. coli* to similar extents, and diversification might occur by acquiring genes and mobile elements not only from other gut-residing bacteria but from (well-established) environmental bacteria as well.

### Spatio-Temporal Dynamics of ESBL-EC During Hospitalization

Considering the nostril swabs that were occasionally found positive for ESBL-EC, we detected the same clone (0–2 SNPs difference) in fecal samples as well, e.g., for horse 69 (t_2_; ST-410 *bla*CTX-M-15) or horse 41 (t_2_, ST10 *bla*CTX-M-1). On the other hand, we also identified a single clone (ST86 *bla*_*SHV*–12_) in nostril swabs of two distinct horses (t_1_, horse 66 and 68), (for details see Supplementary Table 1 and Figures 5A–C). Previous studies which included environmental sampling identified ESBL-EC contamination of horse stables, the floor, medical equipment and other sites as a possible source for in-ho transmission (Walther et al., 2014b; Schoster et al., 2020). While the detection of ESBL-EC in nostril swabs screened at hospital admission was assumed to represent prior contamination, for instance associated with nasogastric intubation (Walther et al., 2018b), sampling results presented here point toward a direct or indirect transmission during hospitalization. These results are in concordance with previous research revealing veterinary clinics as “hotspots” for transmission of MDR Enterobacterales (Wright et al., 2005; Apostolakos et al., 2017; Walther et al., 2018b). Some closely related isolates were able to spread over several months-for example, in the period from January to August 2019 (STC10 and STC86). Last but not least, contact with horses was previously identified as a risk factor for humans to become colonized by ESBL-producing bacteria (Huijbers et al., 2013). Since our results are in line with recent reports from other horse clinics, we are confident that local accumulation and spread together with the presence of vulnerable patients receiving antibiotics and other selective agents are the main drivers for the presented observations. Therefore, increasing awareness for the importance of hygiene is necessary to cope with the current challenges presented by MDR and zoonotic pathogens in horse clinics: As a direct consequence of our study results, hygiene recommendations for horse clinics have been recently published (Gehlen et al., 2020).

## Conclusion

MDR pathogen accumulation in horse clinics, including ESBL-EC, is a threat to both, the equine patients and the people working with and around them. Considering the proverb “an ounce of prevention is worth a pound of cure,” the time to critically re-consider hygiene recommendations and common protocols for the administration of antibiotics in equine medicine appears overdue, especially if prolonged antibiotic courses seem to lack scientific proof of superior outcomes in patients, which needs to be addressed thoroughly in further studies.

## Data Availability Statement

The datasets presented in this study can be found in online repositories. The names of the repository/repositories and accession number(s) can be found in the article/Supplementary Material.

## Ethics Statement

According to the German regulation authorities for research with animal subjects, the comparison of two perioperative prophylaxis regimens in horses subjected to colic surgery does not require approval (Landesamt für Gesundheit und Soziales, Berlin, 18.04.2017). Written informed consent was obtained from the owners for the participation of their animals in this study.

## Author Contributions

BW, AL-B, and HG designed the project. SS, HG, and BW conceived and designed the experiments. TS sequenced the isolates. AK, DK, SS, HG, and AL-B performed laboratory analysis. BW, LE, AL-B, SW, TS, SG, and FG analyzed the data. AK, SW, and BW wrote the article. All authors have read and approved the final draft of the manuscript.

## Conflict of Interest

The authors declare that the research was conducted in the absence of any commercial or financial relationships that could be construed as a potential conflict of interest.

## Footnotes

1 https://github.com/tseemann/snp-dists

2 https://github.com/raivokolde/pheatmap
