# Supplementary figures and images for: Frequency, Local Dynamics, and Genomic Characteristics of ESBL-Producing Escherichia coli Isolated From Specimens of Hospitalized Horses
Source: Front Microbiol. 2021 Apr 16;12:671676. doi: 10.3389/fmicb.2021.671676 (PMC8085565; doi:10.3389/fmicb.2021.671676)

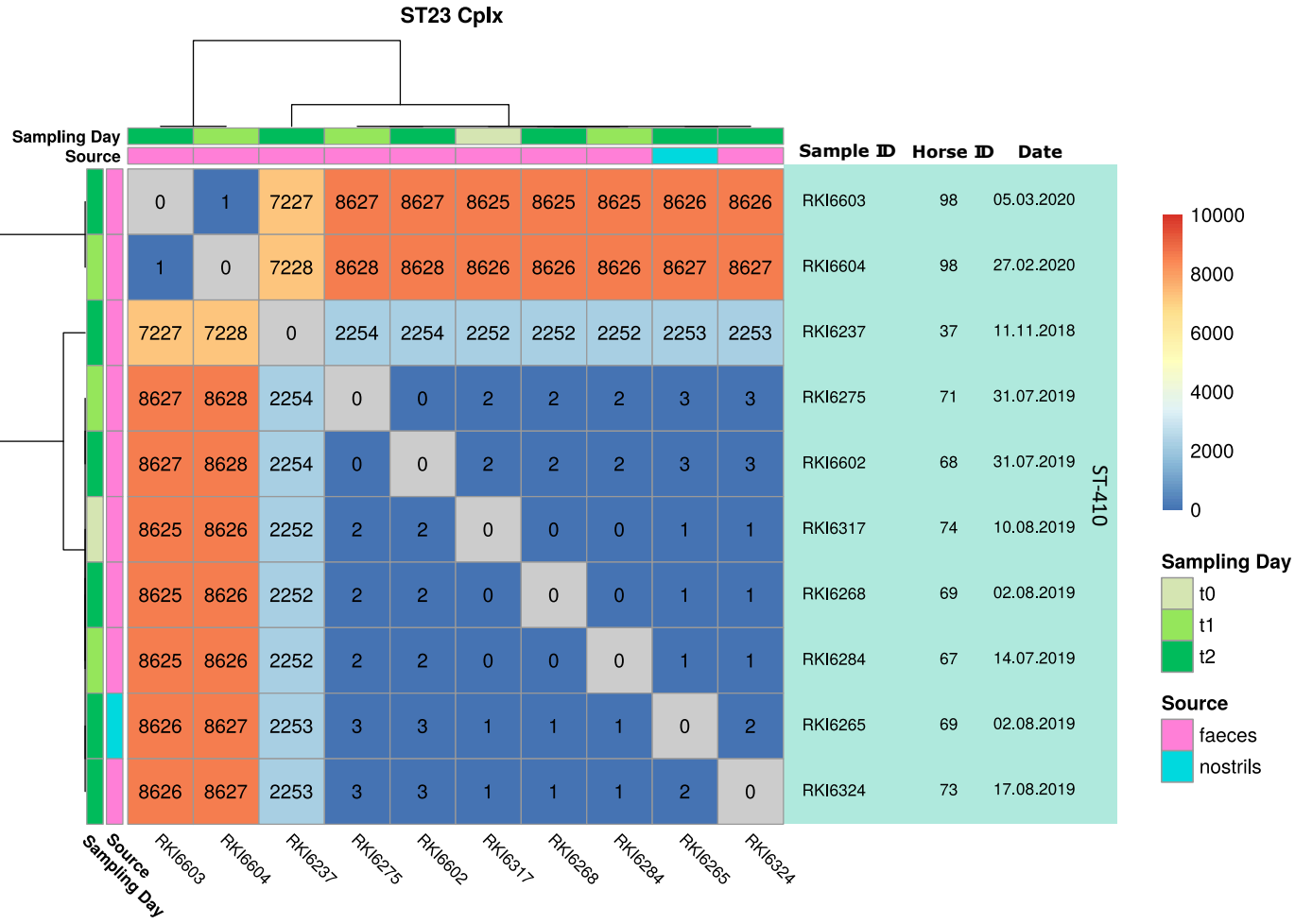

Supplement: Supplementary file 2 [file Data_Sheet_1.PDF]

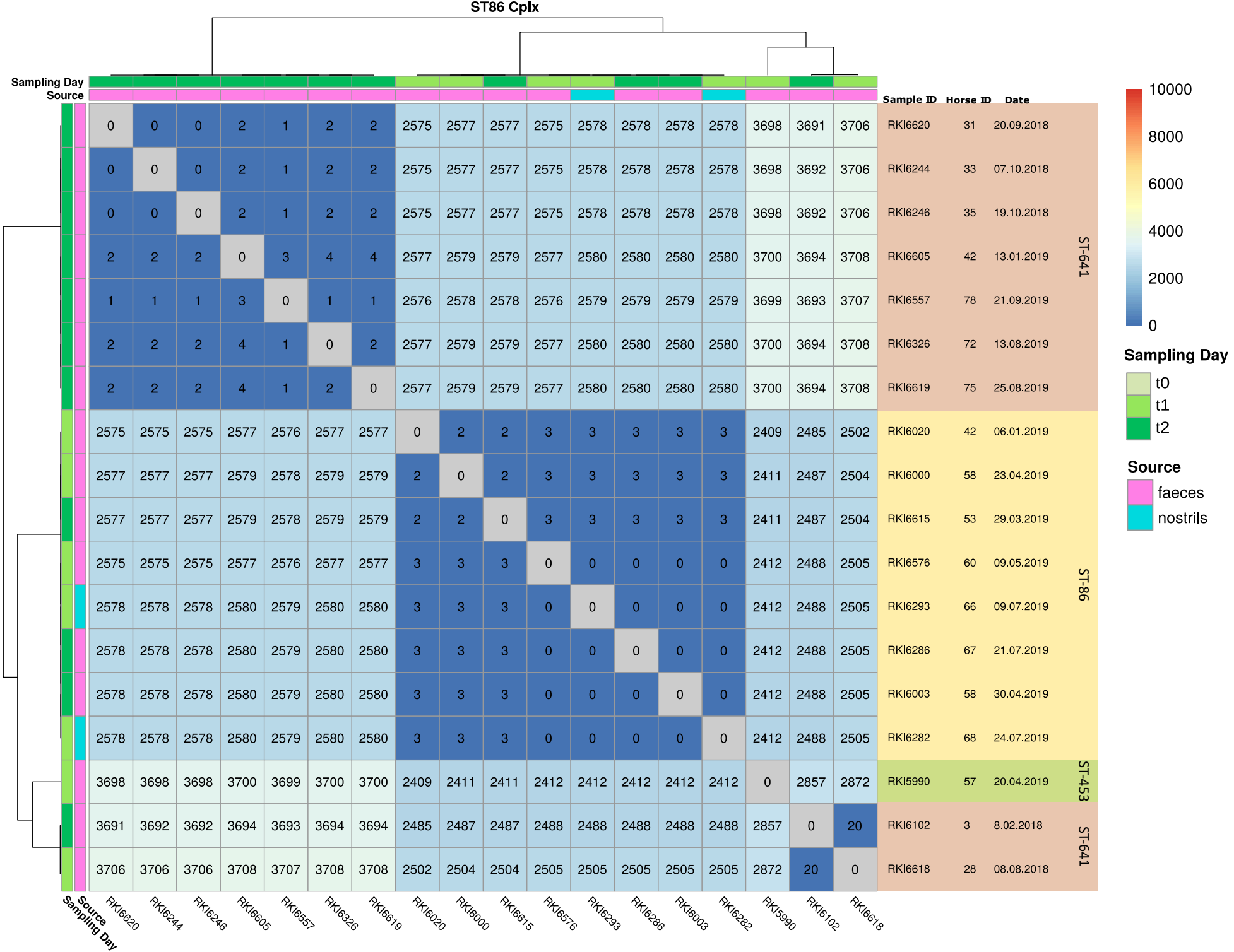

Supplement: Supplementary file 3 [file Data_Sheet_2.PDF]

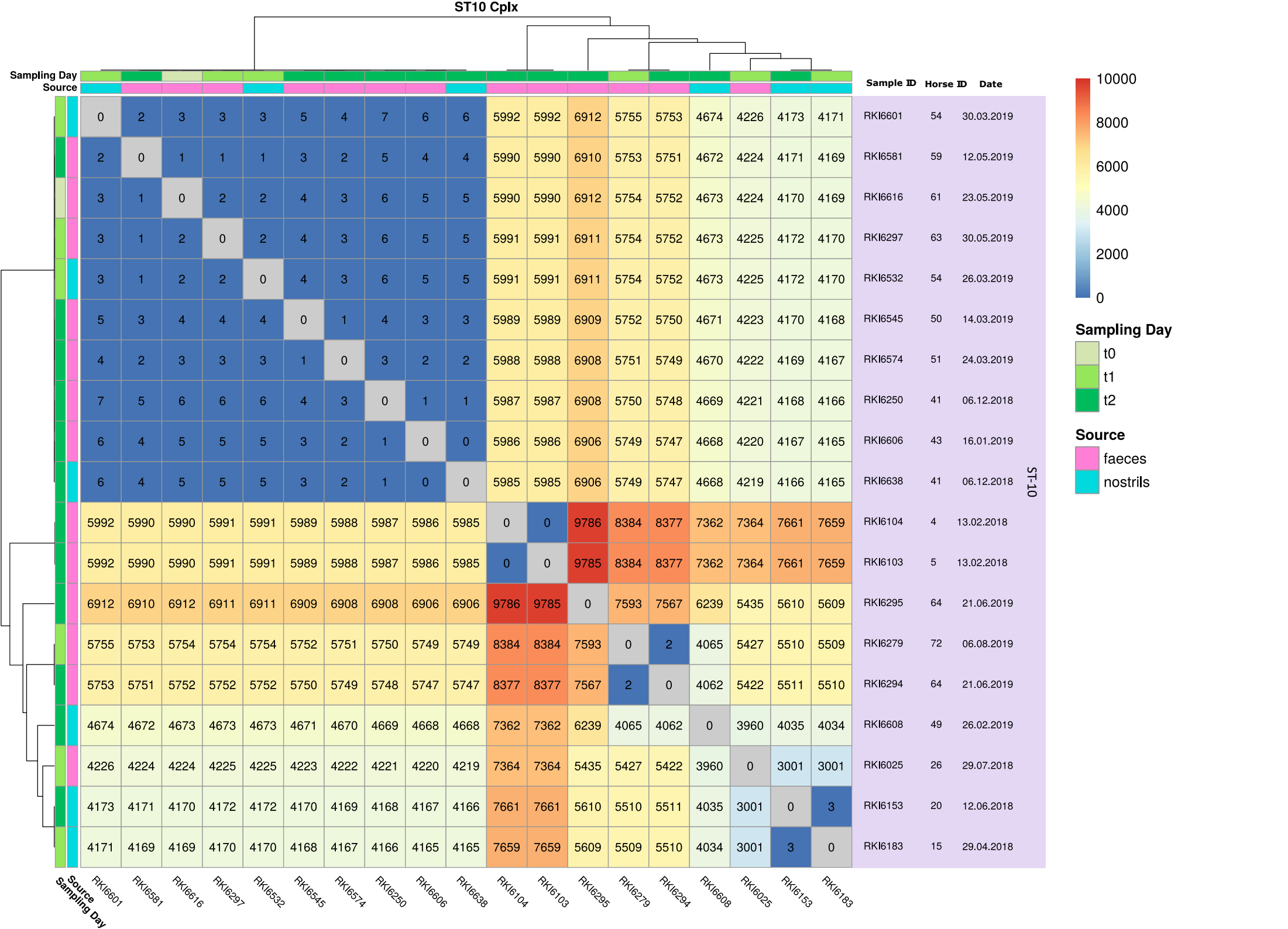

Supplement: Supplementary file 4 [file Data_Sheet_3.PDF]
